# Technological and Enzymatic Characterization of Autochthonous Lactic Acid Bacteria Isolated from *Viili* Natural Starters

**DOI:** 10.3390/foods13071115

**Published:** 2024-04-05

**Authors:** Giorgia Rampanti, Andrea Cantarini, Federica Cardinali, Vesna Milanović, Cristiana Garofalo, Lucia Aquilanti, Andrea Osimani

**Affiliations:** Dipartimento di Scienze Agrarie, Alimentari ed Ambientali, Università Politecnica delle Marche, via Brecce Bianche, 60131 Ancona, Italy; g.rampanti@pm.univpm.it (G.R.); s1107663@studenti.univpm.it (A.C.); v.milanovic@univpm.it (V.M.); c.garofalo@univpm.it (C.G.); l.aquilanti@univpm.it (L.A.); a.osimani@univpm.it (A.O.)

**Keywords:** *viili*, fermented milk, lactic acid bacteria, technological properties, dairy starters

## Abstract

*Viili*, a Finnish ropy fermented milk, is traditionally manufactured through spontaneous fermentation, by mesophilic lactic acid bacteria and yeast-like fungi, or back-slopping. This study evaluated four natural *viili* starters as sources of lactic acid bacteria for dairy production. Back-slopping activation of the studied *viili* samples was monitored through pH and titratable acidity measurements and enumeration of mesophilic lactic acid bacteria. Sixty lactic acid bacteria isolates were collected, molecularly identified, and assayed for acidification performance, enzymatic activities, production of exopolysaccharides (EPSs), presence of the histidine decarboxylase (*hdcA*) gene of Gram-positive bacteria, and production of bacteriocins. A neat predominance of *Lactococcus lactis* emerged among the isolates, followed by *Enterococcus faecalis*, *Enterococcus faecium*, *Enterococcus durans*, *Enterococcus lactis*, and *Lactococcus cremoris*. Most isolates exhibited proteolytic activity, whereas only a few enterococci showed lipase activity. Five isolates identified as *L. cremoris*, *L. lactis*, and *E. faecalis* showed a good acidification performance. Most of the isolates tested positive for leucine arylamidase, whereas only one *E. durans* and two *L. lactis* isolates were positive for valine arylamidase. A few isolates also showed a positive reaction for beta-galactosidase and alpha- and beta-glucosidase. None of the isolates produced EPSs or bacteriocins. The *hdcA* gene was detected in five isolates identified as *L. lactis* and *E. faecium*. A few *L. cremoris* and *L. lactis* isolates for potential use as starter or adjunct cultures for dairy processing were finally identified.

## 1. Introduction

The origin of fermented milks is not exactly defined; however, the production of these dairy products is undeniably intertwined with the domestication of mammals, which facilitated increased access to milk [1]. Over the years, numerous fermented dairy products have emerged worldwide as a mean to preserve milk from spoilage, and nowadays, they represent traditional foods with significant cultural value. However, the development of fermented milk products varied across geographical regions, likely driven by a combination of environmental factors and cultural practices of early farmers [2]. Indeed, the type of milk, the manufacturing practices, and the applied process conditions are the primary factors determining the physico-chemical, microbiological, and sensory properties of these products [3].

The *Codex Alimentarius* defines fermented milk as a milk product obtained by fermentation of milk by the action of suitable microorganisms resulting in a reduction in pH with or without coagulation (iso-electric precipitation of caseins) [4]. Moreover, the *Codex Alimentarius* also specifies that the starter microorganisms shall be viable, active, and abundant in the product to the date of minimum durability [4]. Three categories of fermented milks are generally recognized: (i) thermophilic sour milks, which undergo fermentation at 42–45 °C, resulting in the production of lactic acid; (ii) mesophilic sour milks, where the fermentation is conducted at 20–30 °C, resulting in the production of lactic acid; (iii) acid and alcoholic milks, where the fermentation occurs at 15–25 °C, resulting in the production of alcohol alongside lactic acid and carbon dioxide [5].

Before the introduction of modern refrigeration systems, the evolution of food fermentations strictly depended on climatic conditions; consequently, thermophilic lactic acid fermentations were favored in hot and subtropical regions, whereas mesophilic fermentations prevailed in colder climates [3,6]. Traditionally, fermented dairy products arose from the spontaneous fermentation of milk driven by the autochthonous microbiota. At the present time, the production of fermented milks usually involves the use of starter cultures, which are obtained either from a previous successful manufacture (back-slopping) or through the direct inoculation of selected microbial cultures [7]. The advantages of utilizing commercial starter cultures include enhanced microbial safety and the standardization of the final product. However, a commonly reported concern is the potential loss of biodiversity and the unique characteristics of naturally fermented products [8,9]. Indeed, artisanal fermented dairy products constitute ecosystems with a high microbial diversity that should be preserved [10,11,12]. This has driven research into the characterization of complex microbial communities to gain deeper insights into their species composition and functions [13]. Nevertheless, the use of artisanal starters in the development of fermented dairy products necessitates evaluating the safety and technological traits of the selected cultures [14,15,16,17].

In Northern European countries (e.g., Denmark, Finland, Iceland, Norway, and Sweden), milk and dairy products have historically been considered as essential components of the human diet [9]. This was due to the constraints on food crop cultivation imposed by climatic conditions in these regions [9]. As a result of the necessity for dietary diversity, a wide range of fermented dairy products has been developed. These include cultured creams, buttermilks, cultured milks, cultured buttermilks, ropy fermented milks, and concentrated fermented milks [9,18]. Among them, ropy fermented milk products, such as the Finnish *viili*, the Norwegian *tettemelk*, and the Swedish *långfil*, are noted for their distinctive slimy texture, which is attributed to the presence of exopolysaccharides (EPSs) produced by specific microbial strains occurring in the starter culture, primarily *Lactocococcus lactis* subsp. *cremoris* [9]. Interestingly, in Scandinavian folklore, the production of ropy fermented milks was obtained through the addition of leaves from the carnivorous plant *Pinguicula vulgaris* to milk [9]. Although this practice is no longer used, research by Porcellato et al. [19] has revealed the presence of *Lactococcus* spp. (carrying the EPS operon) in the microbiome of *P. vulgaris*.

Alongside *L. lactis* subsp. *cremoris*, the presence of other lactic acid bacteria species has also been documented in Nordic ropy fermented milks [20,21]. In more detail, Chen et al. [20] isolated the species *Lactobacillus plantarum*, *Streptococcus thermophilus*, *Lactobacillus paracasei*, and *Lactobacillus delbrueckii* from artisanal *viili*, whereas Kahala et al. [21] reported the isolation of *L. lactis* subsp. *lactis*, *L. lactis* subsp. *lactis* biovar *diacetylactis*, and *Leuconostoc mesenteroides* from industrial *viili*. Moreover, the yeast-like fungus *Geotrichum candidum* is known to commonly grow on the surface layer of this product [9]. However, most of the research on *viili* focuses on its health benefits associated with the presence of EPSs [22,23].

Ropy fermented milk products are commercially available in Nordic countries [9]. However, the use of commercial starter cultures in large-scale manufacturing may result in products that deviate from traditional ones [9]. Based on these premises, the objective of the present study was to isolate, identify, and characterize lactic acid bacteria from *viili* dry natural starters with the aim of evaluating the potential use of these microorganisms as starter or adjunct cultures for *viili* production resembling artisanal methods, as well as exploring their broader applications within the dairy industry.

Therefore, 60 lactic acid bacteria isolates were molecularly identified and characterized for (i) acidification performance; (ii) key enzymatic activities; (iii) production of sucrose-dependent and -independent EPSs; (iv) presence of the *hdcA* gene of Gram-positive bacteria encoding for histidine decarboxylase; and (v) production of bacteriocins against *Listeria innocua*, used as surrogate for *Listeria monocytogenes* [24].

## 2. Materials and Methods

### 2.1. Activation of Viili Starters and Monitoring of Fermentation

Four commercially available *viili* dry natural starters, denoted as V1, V2, V3, and V4, were activated according to the protocol described by Chen et al. [20]. Briefly, the starters were incubated at a concentration of 5% (w v^−1^) in sterilized bovine milk at 25 °C for 20 h. Subsequently, the inoculated milk was transferred into fresh sterilized milk under the same conditions. After three additional sub-culturing steps, the starters were considered active. No information on the production process of the starters was provided by the suppliers.

The fermentation process was monitored at each sub-culturing stage by measuring pH and titratable acidity, as detailed by Rampanti et al. [25]. Furthermore, plate counting of viable mesophilic lactic acid bacteria on M17 agar (Liofilchem, Roseto degli Abruzzi, Italy) incubated at 30 °C for 48 h was performed. At the end of the activation process, the content of lactic and acetic acid was determined using the D-/L-Lactic Acid (D-/L-Lactate) (Rapid) test kit and the Acetic Acid (Acetate Kinase Manual Format) test kit (Megazyme, Bray, Ireland), respectively. The results were expressed as mean ± standard deviation of three independent measurements.

### 2.2. Isolation and Characterization of Lactic Acid Bacteria

For each *viili* production performed using the dry natural starters, colonies grown on M17 agar were randomly selected and subsequently sub-cultured to purity under the same conditions. The obtained bacterial isolates were stored at −80 °C in a 1:1 sterile mixture of M17 broth and glycerol (50% in water) for long-term storage.

Cryopreserved bacteria were sub-cultured twice and subjected to DNA extraction according to Osimani et al. [26]. DNA was checked for quantity and purity with a NanoDrop ND 1000 (Thermo Fisher Scientific, Wilmington, DE, USA), standardized to a final concentration of 100 ng µL^−1^, and subjected to PCR in a Mastercycler X50a Thermocycler (Eppendorf, Hamburg, Germany) using the universal prokaryotic primers 27f and 1495r, as described by Osimani et al. [26]. The amplicons were then shipped to Genewiz (Leipzig, Germany) for purification and sequencing. The obtained sequences were compared to 16S rRNA sequences of type strains from the GenBank DNA database (http://www.ncbi.nlm.nih.gov/) (accessed on 14 November 2023) through the basic local alignment search tool (BLAST). Finally, lactic acid bacteria cultures were submitted to the GenBank DNA database to obtain accession numbers.

### 2.3. Enzymatic Activities

The enzymatic activities of lactic acid bacteria were evaluated by agar assays according to Linares-Morales et al. [27] with some modifications. Prior to the test, the isolates were retrieved from the frozen-stored suspensions and sub-cultured twice on the same medium used for the isolation. For each isolate, 5 µL of the overnight culture was spotted in triplicate on the following media: (i) skim milk agar (peptone 0.5%, malt extract 0.3%, yeast extract 0.3%, glucose 1%, sodium chloride 0.5%, agar 0.2%, skim milk 2%) for the evaluation of protease activity; (ii) tributyrin agar (peptone 0.5%, yeast extract 0.3%, agar 2%, tributyrin 1%) for lipase activity; (iii) tween 80 agar (peptone 1%, sodium chloride 0.5%, calcium chloride 0.01%, agar 0.2%, tween 80 1%) for esterase activity. The presence of an opaque precipitate (esterase activity) or a clear halo (protease and lipase activity) around the inoculum after incubation at 30 °C for 48 h indicated a positive result, with intensity levels denoted as + (1 mm), ++ (1–2 mm), and +++ (>2 mm).

**Figure 1:**
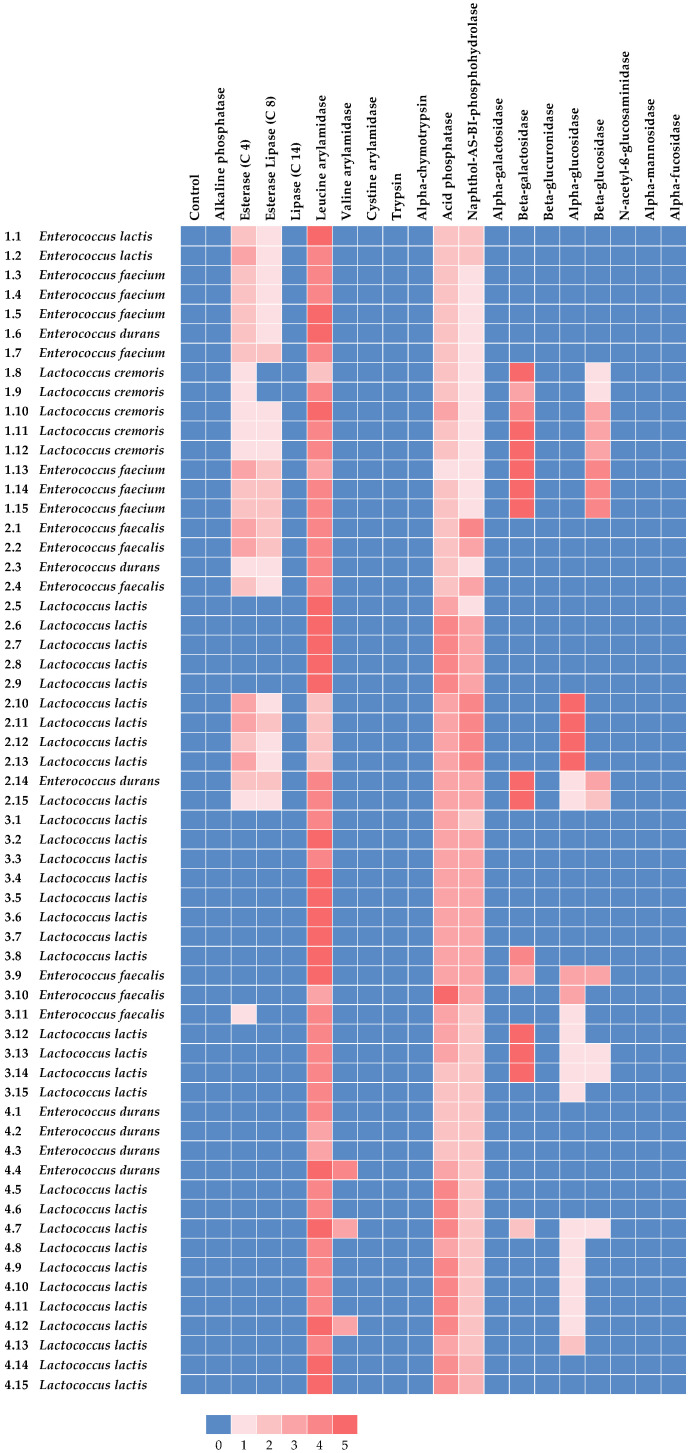
Semi-quantitative assessment of enzymatic activity of lactic acid bacteria isolated from *viili* samples.

To acquire further insight into enzymatic activities, the isolates were also tested using the semi-quantitative, color-based, micromethod API^®^ ZYM (bioMérieux, Marcy l’Etoile, France) according to the manufacturer’s instruction. API^®^ ZYM galleries are designed to determine the reactions of the enzymes listed in Figure 1. After color development (~5 min), a value ranging from 0 to 5 was assigned: 0, corresponding to a negative reaction; 1, 2, 3, 4, intermediate reactions depending on the level of intensity (3, 4, or 5 being considered as a positive reaction); 5, maximum intensity.

### 2.4. Acidification Performance

The acidification performance of the isolates was assessed as previously detailed by Nicosia et al. [16] with few modifications. First, the isolates were retrieved from the frozen-stored suspensions and sub-cultured twice on the same medium used for the isolation. After growth, bacterial suspensions were centrifuged (1610× *g* for 5 min), and the cell pellets were resuspended in physiological solution (NaCl 0.9% w v^−1^) to reach a turbidity level equivalent to 1 McFarland unit. The resulting suspension was inoculated in triplicate for each isolate at a concentration of 2% (v v^−1^) into sterile reconstituted skim milk (10% w v^−1^). The acidification was determined by measuring the pH prior to inoculation (t0) and after 6, 8, and 24 h of incubation at 30 °C using a pH meter equipped with a HI2031 solid electrode (Hanna Instruments, Padova, Italy).

### 2.5. In Vitro EPS Production

For detecting in vitro EPS-producing isolates, the method previously described by Hilbig et al. [28] was used with few adjustments. First, the isolates were retrieved from the frozen-stored suspensions and sub-cultured twice at 30 °C for 48 h. Then, 5 µL of each bacterial culture was spotted in triplicate on the following media: MRS agar added with sucrose at a concentration of 80 g L^−1^ to promote the synthesis of homopolysaccharides (HoPSs); MRS agar added with yeast extract (VWR Chemicals) (10 g L^−1^), meat extract (VWR Chemicals) (10 g L^−1^), lactose (Carlo Erba, Cornaredo, Italy) (20 g L^−1^), and galactose (VWR Chemicals) (20 g L^−1^) to promote the synthesis of heteropolysaccharides (HePSs). After 48 h incubation at 30 °C, colonies were classified as positive if they showed either a ropy consistency (able to form visible filaments with a sterile toothpick) or a mucoid appearance (displaying a visible glossy and slimy texture).

### 2.6. Detection of the hdcA Gene of Gram-Positive Bacteria

The isolates were tested for the presence of the histidine decarboxylase (*hdcA*) gene through qPCR performed using a CFX Connect Real-Time System machine (BioRad, Hercules, CA, USA), following the cycling conditions and primers previously described by Belleggia et al. [29] for the amplification of a 174 bp fragment of the *hdcA* gene [30]. The positive strain *Lactobacillus parabuchneri* DSM 5987 was used to create the standard curve. The analysis was performed in triplicate for each isolate, together with a blank. The results were expressed as the presence (+) or absence (−) of the target gene in the bacterial DNA samples.

### 2.7. Assessment of Antimicrobial Activity

The antimicrobial activity was assessed through the agar well diffusion assay as previously described by Cardinali et al. [31]. Prior to the test, the isolates were retrieved from frozen-stored suspensions and sub-cultured twice at 30 °C for 48 h; a third sub-culturing step was performed for 24 h. After the collection of an aliquot (500 µL) of the bacterial culture, the remaining broth cultures were centrifuged at 1610× *g* for 10 min. Then, the supernatant was neutralized (pH 7) with 0.1 N NaOH (AppliChem, Darmstadt, Germany). A filtration step on a sterile PES membrane filter of 0.22 μm pore size (Laboindustria S.p.A., Padova, Italy) was also performed. The Brain Heart Infusion (BHI) (VWR) soft agar (0.75%) growth medium was inoculated at a concentration of 2% (v v^−1^) with the target microorganism *Listeria innocua* and subsequently poured into a 90 mm diameter Petri dish. After solidification, a 200 µL sterile tip cone was used to create wells of ~50 µL. For each isolate, 4 wells were formed containing the following: (i) 50 μL of the sub-cultured suspension; (ii) 50 μL of the neutralized suspension; (iii) 50 μL of the filtered neutralized suspension; (iv) 50 μL of sterilized water as negative control. Finally, the Petri dishes were incubated at 37 °C for 24 h and checked for the presence of inhibition halos.

### 2.8. Statistical Analysis

Significant differences among fermented milks inoculated with different *viili* natural starters were determined by one-way analysis of variance (ANOVA) using the software JMP^®^ Version 11.0.0 (SAS Institute Inc., Cary, NC, USA) and the Tukey–Kramer Honest Significant Difference (HSD) test (α = 0.05).

## 3. Results and Discussion

### 3.1. Physico-Chemical and Microbiological Parameters

Over time, food fermentation techniques have evolved from the back-slopping method, where portions of fermented substrates containing high microbial populations were added to raw materials to initiate subsequent fermentations. This method facilitated the selection of autochthonous microorganisms naturally adapted to specific fermentation conditions [32]. However, the microbial populations within natural starter cultures may exhibit variability in both load and species composition. Hence, in the present study, the fermentation of the four *viili* samples obtained using natural dry starters was monitored through physico-chemical measurements and viable counting of mesophilic lactic acid bacteria.

Table 1 provides the parameters monitored during the activation of the studied *viili* dry starters. The pH values of milk soon after inoculation ranged from 6.35 ± 0.07 (V2) to 6.55 ± 0.07 (V4), with no statistically significant difference among samples. Concurrently, the samples showed low levels of titratable acidity (approximately 0.14% of lactic acid equivalents). At t_0_, V3 showed the highest load of presumptive lactic acid bacteria (3.95 ± 0.11 Log cfu g^−1^), followed by V4 (3.26 ± 0.02 Log cfu g^−1^), V2 (2.68 ± 0.23 Log cfu g^−1^), and V1 (1.85 ± 0.00 Log cfu g^−1^). After 20 h of incubation at 30 °C (t_1_), a significant increase in the load of presumptive lactic acid bacteria was observed in all samples, ranging from 6.29 ± 0.06 (V2) to 9.25 ± 0.04 Log cfu g^−1^ (V3). At the same sampling time (t_1_), V3 showed statistically lower pH and higher titratable acidity values compared to the other samples. Conversely, higher pH and lower titratable acidity were observed in V1 and V2, whereas V4 showed intermediate values. All samples reached counts of presumptive lactic acid bacteria of approximately 9 Log cfu g^−1^ after the first sub-culturing step (t_2_). However, statistically significant differences for pH and titratable acidity were observed among samples. Specifically, V3 and V4 showed lower pH and higher titratable acidity (approx. 4.32 and 0.75%, respectively) compared to V1 and V2 (approx. 5.79 and 0.25%, respectively). In V2, V3, and V4, the load of presumptive lactic acid bacteria remained stable until the end of the activation process. However, V1 showed statistically lower counts compared to the other samples at t_2_, t_3_, and t_4_, ranging from 8.49 to 8.99 Log cfu g^−1^. Although the pH and titratable acidity in V1 were also statistically different at t_3_ compared to the other samples, no differences were observed after the last sub-culturing step (t_4_). Indeed, at this sampling time, pH and titratable acidity ranging from 4.27 to 4.40 and 0.73 to 0.76, respectively, were found in all samples. As for organic acids at the end of the activation process, V4 showed lower values of both lactic and acetic acid compared to the other samples (1.54 ± 0.27 and 0.06 ± 0.06 g 100 g^−1^, respectively). No statistically significant differences were found for lactic acid among V1, V2, and V3, with values ranging from 2.79 to 2.97 g 100 g^−1^. Moreover, V3 showed the highest acetic acid content, 0.63 ± 0.02 g 100 g^−1^, followed by V1 (0.48 ± 0.04 g 100 g^−1^) and V2 (0.24 ± 0.03 g 100 g^−1^).

The result obtained showed that the proper activation of the studied *viili* samples was then reached.

### 3.2. Identification of the Lactic Acid Bacteria Isolates

The alignment results of the 16S rRNA sequences obtained from 60 lactic acid bacteria isolated from the *viili* samples allowed their unambiguous identification to be achieved. The closest relatives, the percent identities, and the accession numbers of the obtained sequences are reported in Table 2.

In more detail, lactococci represented the most frequently isolated lactic acid bacteria (38 isolates), with *Lactococcus lactis* being the most isolated species (33 isolates), followed by *Lactococcus cremoris* (5 isolates).

Moreover, *Enterococcus faecium* (seven isolates), *Enterococcus durans* (seven isolates), *Enterococcus faecalis* (six isolates), and *Enterococcus lactis* (two isolates) were also isolated and identified.

*Lactococcus* spp., including *L. lactis* and *L. cremoris*, have previously been found among the predominant isolates in *viili* samples [21]. Of note, this lactic acid bacteria genus is widely associated with the milk environment and constitutes the primary component in the starter culture used to produce both artisanal and industrial dairy products worldwide [33,34].

On the contrary, to the best of the authors’ knowledge, this is the first research revealing the presence of *Enterococcus* spp. in *viili* and, more generally, in Nordic ropy fermented milks. However, enterococci have previously been isolated in other traditional fermented milks, such as *Gariss* and *Shubat* (obtained from camel milk) [35], *Gioddu* (obtained from ovine or goat milk) [36], and *kumis* (obtained from bovine milk) [37]. Among enterococci, the species *E. faecium*, *E. faecalis*, and *E. durans* are the most frequently found in cheese [38]. The occurrence of enterococci in fermented foods, particularly those of animal origin such as milk and dairy products, can be attributed to their association with the mammalian gastrointestinal tract and their ability to resist and grow in adverse extra-enteric environments [39]. Although enterococci have been demonstrated to have positive effects in fermented foods (e.g., enhancement of sensory properties, production of bacteriocins, and potential probiotic functions), they do not possess the Qualified Presumption of Safety (QPS) status in the EU and are not Generally Recognized as Safe (GRAS) in the USA [38]. In fact, their ability to disseminate virulence and antibiotic-resistance genes poses potential risks to consumers’ health that deserve further investigation [38].

### 3.3. Enzymatic Activities of the Lactic Acid Bacteria Isolates

The results of the assessment of protease, lipase, and esterase activities of the lactic acid bacteria isolated from the *viili* samples are reported in Table 3. In more detail, most of the isolates (43 out of 60) showed a positive reaction for protease activity. Remarkably, isolates 1.3, 1.7, 1.12, 2.11, 2.14, 2.15, and 4.15 exhibited higher protease activity. Of note, almost all isolates belonging to the enterococci species showed protease activity, whereas higher variability was observed for lactococci. The proteolytic system of lactic acid bacteria consists of three main components: (i) cell-wall proteinase, which hydrolyzes extracellular caseins; (ii) peptide transporters, responsible for carrying oligopeptides into the cell; and (iii) intracellular peptidases, which break down peptides into shorter peptides and amino acids [40].

These mechanisms are essential for cells’ survival and multiplication during fermentation as they enable the supply of amino acids necessary for bacterial growth. Furthermore, the proteolysis of lactic acid bacteria is regarded as a crucial technological property in the dairy sector because it is associated with the development of volatile compounds that can affect the flavor of the end product [41,42]. Moreover, the proteolytic activity of lactic acid bacteria can provide potential health benefits for consumers, including improved digestibility and the release of bioactive peptides [41,42]. In general, lactic acid bacteria are weakly proteolytic compared to other microbial groups found in the microbiota of dairy products [43]. However, this property may vary depending on the species, the subspecies, and even the specific strain [44]. *Lacticaseibacillus casei*, *Lactobacillus delbrueckii* subsp. *bulgaricus*, *Lactobacillus helveticus*, and *Lactobacillus acidophilus* are the lactic acid bacteria that, due to their proteolytic activity, are the most used in the dairy industry. Among lactococci, *L. cremoris* typically exhibits higher proteolytic activity compared to *L. lactis* [44]. Moreover, the proteolytic activity of *Enterococcus* spp. is well documented in the literature [27,45,46,47].

In the present study, only 11 isolates (1.1, 1.5, 1.13, 1.14, 1.15, 2.1, 2.2, 2.14, 4.1, 4.2, 4.4) showed lipase activity, whereas no isolate exhibited esterase activity. Notably, all the isolates testing positive for lipolytic activity belonged to the enterococci group. Lipases are enzymes that catalyze the hydrolysis of triglycerides to glycerol and free fatty acids over an oil–water interface, whereas esterase catalyzes the hydrolysis of esters in solution and tri-, di-, and monoglycerides containing short-chain fatty acids [48,49]. Lactic acid bacteria are known to be weakly lipolytic compared to other microbial groups [49]. Thus, the results of this research are in accordance with those usually reported in the scientific literature. Indeed, the lipolytic activity of *Lactococcus* spp. isolated from dairy products has rarely been reported [16,50,51,52]. Instead, there is more evidence on the lipolytic activity of *Enterococcus* spp. [47,53,54].

The results of the semi-quantitative assessment of the enzymatic activities by API^®^ ZYM are reported in Figure 1. In more detail, none of the isolates exhibited a positive reaction for alkaline phosphatase, lipase (C 14), cystine arylamidase, trypsin, alpha-chymotrypsin, alpha-galactosidase, beta-glucuronidase, N-acetyl-ß-glucosaminidase, alpha-mannosidase, and alpha-fucosidase. Conversely, all isolates showed leucine arylamidase, acid phosphatase, and naphthol-AS-BI-phosphohydrolase activities.

As for leucine arylamidase, all isolates except *L. cremoris* 1.8 and *L. lactis* 2.10, 2.11, 2.12, and 2.13 exhibited strong activity for this enzyme. The activity of leucine arylamidase in both *Lactococcus* spp. and *Enterococcus* spp. is widely documented in the literature [55,56,57] and considered as a desirable trait for dairy starters [55]. Conversely, the activity of other aminopeptidases is less frequently detected [57,58]. Of note, isolates *E. durans* 4.4 and *L. lactis* 4.7 and 4.12 exhibited a positive reaction for valine arylamidase. Weak esterase (C 4) and esterase lipase (C 8) activities were observed for a few isolates; however, only isolates *E. lactis* 1.2; *E. faecium* 1.13; *E. faecalis* 2.1, 2.2; and *L. lactis* 2.10, 2.11, 2.13 exhibited a positive reaction for esterase (C 4).

In the scientific literature, high esterase (C 4) and esterase lipase (C 8) activities are usually reported for enterococci [59,60,61]. Moreover, the esterase (C 4) activity of *L. lactis* has been already observed by Abarquero et al. [56] and Kamarinou et al. [58] in strains isolated from artisanal cheeses.

As for acid phosphatase, the activity of this enzyme may influence the hydrolysis of caseins in dairy products [62]. Except for isolate *L. cremoris* 1.10, all isolates of the same species sourcing from V1 exhibited a negative reaction to acid phosphatase. The same result was also observed for isolates 2.1, 2.2, 2.3, 2.4, 3.14, 3.15, 4.1, 4.2, and 4.3. Conversely, isolates 2.6, 2.7, 2.8, 2.9, 4.5, 4.6, 4.7, 4.9, 4.10, 4.11, and 4.12, all belonging to the *L. lactis* species, showed a strong reaction to acid phosphatase. Moreover, *E. faecalis* 3.10 was the sole isolate showing the strongest reaction to acid phosphatase. Of note, the acid phosphatase activity of *Enterococcus* spp. (*E. durans*, *E. faecium*, and *E. faecalis*) and *L. lactis* has previously been found in strains isolated from artisanal cheeses [56,60].

None of the isolates originating from V1 and V4 showed a positive naphthol-AS-BI-phosphohydrolase reaction. Conversely, except for 2.3, 2.5, 3.1, 3.11, 3.12, 3.13, 3.14, and 3.15, a positive reaction was observed for the other isolates. Similarly to acid phosphatase, the action of naphthol-AS-BI-phosphohydrolase is associated with potential technological and health benefits [63].

Few isolates exhibited positive reactions for beta-galactosidase, alpha-glucosidase, and beta-glucosidase, which are enzymes involved in sugar metabolism. In more detail, isolates *L. cremoris* 1.8, 1.9, 1.10, 1.11, 1.12, *E. faecium* 1.13, 1.14, 1.15, *E. durans* 2.14, *L. lactis* 2.15, 3.8, 3.12, 3.13, 3.14, and *E. faecalis* 3.9 exhibited a positive reaction for beta-galactosidase. This enzyme hydrolyzes the lactose beta-glycosidic bond between glucose and galactose [64], thereby holding significant importance in both technological and nutritional aspects. In fact, the activity of beta-galactosidase may contribute to milk acidification and the alleviation of lactose intolerance for consumers [65]. The beta-galactosidase activity exhibited by *Enterococcus* spp. is extensively documented in the literature, thus confirming their adaption to the dairy environment [57,59,60]. Of note, a low beta-galactosidase activity exhibited by *Lactococcus* spp. isolates was observed by Abarquero et al. [56]. In the present study, only the isolates *L. lactis* 2.10, 2.11, 2.12, and 2.13 and *E. faecalis* 3.9 and 3.10 showed alpha-glucosidase activity. Moreover, beta-glucosidase activity has also rarely been detected. In fact, only isolates *L. cremoris* 1.10, 1.11, 1.12, *E. faecium* 1.13, 1.14, 1.15, *E. durans* 2.14, and *E. faecalis* 3.9 exhibited a positive reaction for this enzymatic activity. Beta-glucosidase catalyzes the hydrolysis of terminal residues of beta-D-glucose [66] and has scarcely been found among the enzymatic activities of lactic acid bacteria isolated from dairy products [61,67,68].

It is noteworthy that all the isolates showed the absence of beta-glucuronidase activity, which is associated with the development of carcinogenic compounds in the human colon [31].

The assessment of enzymatic activities of lactic acid bacteria represents a valuable tool for the initial screening of potential starter or adjunct cultures in the dairy industry. Indeed, the metabolic reactions of microorganisms significantly impact the nutritional and sensory (volatile) properties of fermented foods [16,47,69]. However, criteria for selecting starter or adjunct cultures with specific technological properties may vary depending on the type of product. The collected results can provide a preliminary basis for future research aimed at further investigating the isolates’ capacity to produce aromatic or beneficial compounds in dairy products.

### 3.4. Acidification Performance

**Figure 2:**
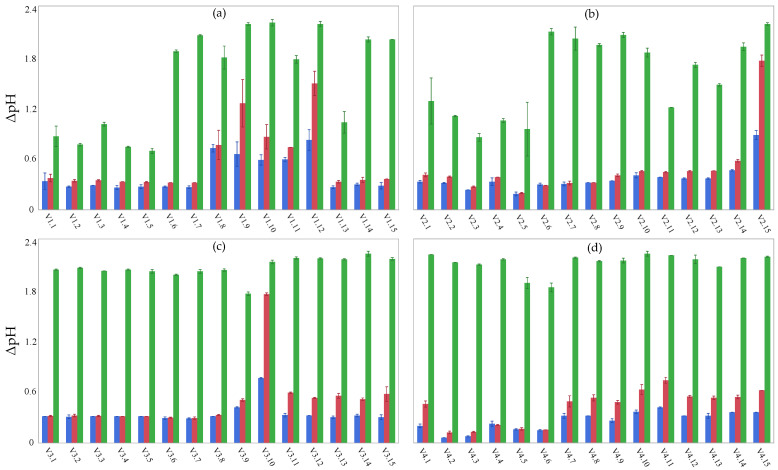
Acidification performance of the lactic acid bacteria isolated from *viili* samples after 6 h (blue bar), 8 h (red bar), and 24 h of fermentation (green bar). Panel (**a**), isolates from V1; panel (**b**), isolates from V2; panel (**c**), isolates from V3; panel (**d**), isolates from V4.

Based on their acidification performance, lactic acid bacteria are commonly classified into three categories: (i) fast acidifiers (pH < 5.3 after 6 h), medium acidifiers (pH < 5.3 after 8 h), and slow acidifiers (pH > 5.3 after 8 h) [16]. The results of the acidification performance of lactic acid bacteria isolated from the *viili* samples studied herein are reported in Figure 2 (V1, panel a; V2, panel b; V3, panel c; V4, panel d).

In more detail, none of the tested isolates lowered the pH of milk below 5.3 after 6 h of fermentation. The ΔpH after 6 h of fermentation ranged from 0.06 to 0.83. Notably, isolates *L. cremoris* 1.8, 1.9, 1.12, *L. lactis* 2.15, and *E. faecalis* 3.10 showed the best acidification performance after 6 h of fermentation. The ΔpH after 8 h of fermentation ranged from 0.12 to 1.73. A pH < 5.3 was measured in milk inoculated with the isolates 1.12, 2.15, and 3.10 after 8 h of fermentation. Moreover, isolate 1.9 also showed a good performance after 8 h of fermentation, resulting in a pH value of 5.33. The other isolates showed a slower acidification rate. However, except for isolates 1.1, 1.2, 1.3, 1.4, 1.5, 1.13, 2.2, 2.3, 2.4, 2.5, and 2.11, all isolates lowered the pH below 5.3 after 24 h of fermentation. The ΔpH after 24 h ranged from 0.65 to 2.20; at this time point, most of the isolates lowered the pH to values around 4.6, thus leading to milk coagulation.

The acidification performance is considered a primary criterion for selecting starter cultures with potential dairy applications [70]. Indeed, rapid fermentation and subsequent acidification are essential for ensuring product safety and texture [71,72]. In more detail, the well-known gel structure of fermented milk products is the result of acidic coagulation of caseins (namely αS1, αS2, β, and κ-casein) that is obtained at a pH of around 4.8, thus strongly affecting the rheological properties of the end product [73]. As reported by Beresford et al. [74], optimal starter bacteria for dairy purposes are expected to produce sufficient acid to lower the pH of milk to below 5.3 in 6 h. Cogan et al. [75] observed that mesophilic lactic acid bacteria exhibited lower acidification activity compared to thermophilic bacteria. *Lactococcus* spp. isolated from dairy products generally show weak acidification activity [16,76,77]; however, strain-specific variations may be observed [78]. Concerning the isolates belonging to enterococci species, the results obtained in the present study are consistent with those previously reported by Serio et al. [60].

### 3.5. In Vitro EPS Production

None of the tested isolates exhibited in vitro EPS production, whether homopolysaccharides (HoPSs) or heteropolysaccharides (HePSs) (Table 3).

EPSs are biopolymers produced by specific microorganisms by extracellular synthesis or secretion during their growth [79]. The presence of EPSs positively influences the rheological and sensory characteristics of fermented dairy products, such as viscosity, syneresis, and sensory properties. Moreover, EPSs may exhibit various functional features, including antioxidant, antibacterial, cholesterol-lowering, immunoregulatory, antitumor, and anticoagulant activities [80]. Consequently, there has been a growing interest in recent years in the utilization of EPS-producing lactic acid bacteria in fermented foods to enhance product quality.

**Figure 3:**
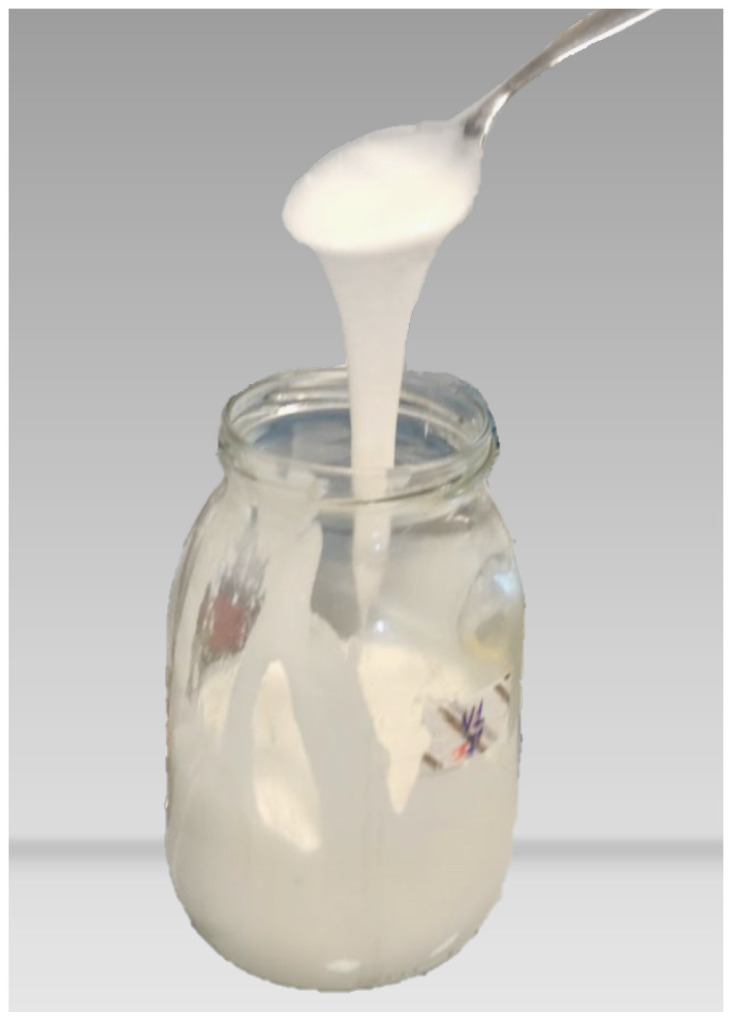
Ropy consistency of *viili* after the activation of natural starters.

The distinctive feature of *viili*, along with other Nordic fermented milks, is their slimy texture, which arises from the production of EPSs by certain species of *Lactococcus*, mainly *L. cremoris* and *L. lactis* [9]. The in vitro assay for testing EPS-producing cultures applied in the present study represents a rapid and useful method for the preliminary selection of lactic acid bacteria as potential adjunct cultures [81]. However, this assay should be complemented by the molecular detection of EPS-related genes for further validation [28,82]. In the present study, the *viili* fermented milk samples obtained at the end of fermentation exhibited the typical ropy texture (Figure 3), thus suggesting the presence of EPSs in the end product.

It is noteworthy that EPS production by lactic acid bacteria depends on several factors, including genetic variation, environmental conditions, and strain-specific differences [83]. In the present study, the in vitro tests did not reveal EPS formation on agar plates, thus indicating the need for further in vivo tests of the isolates (e.g., use of pure cultures inoculated in milk).

### 3.6. Presence of hdcA Gene and Assessment of Antimicrobial Activity

The presence of the *hdcA* gene was revealed by qPCR in isolates *E. faecium* 1.5 and *L. lactis* 2.5, 2.8, 3.13, and 4.14 (Table 3). The gene cluster including the *hdcA* gene encodes for the activity of histidine decarboxylase, responsible for histamine production [84]. Of note, histamine represents a food hazard that is the causative agent of a wide range of foodborne diseases, including headache, hypotension, and vomiting [85]. Cheese and fish are the food categories mostly associated with histamine intoxication [86]. However, histamine content in dairy products is not subjected to any regulation by the European Union [87].

The detection of histamine-producing lactic acid bacteria represents a preventive strategy to avoid their use as starter or adjunct cultures, thus protecting the health of consumers [88,89]. Of note, histamine-producing *L. lactis* strains have already been detected by Roig-Sagués et al. [90] in traditional Spanish cheese. Moreover, Park et al. [91] and Tham et al. [92] previously reported the presence of histamine-producing *Enterococcus* spp. in goat cheese and in *Cheonggukjang* (a traditional Korean food made by fermenting soybeans), respectively.

Another safety-related aspect of interest to consider when selecting lactic acid bacteria to be used as starter or adjunct cultures is their antimicrobial activity against spoilage or pathogenic bacteria [88]. Indeed, some lactic acid bacteria have the capability to produce antimicrobial peptides, known as bacteriocins, which exhibit inhibitory activities against foodborne pathogens or spoilage microorganisms [93]. In more detail, strains of *Lactococcus* spp. and *Enterococcus* spp. isolated from dairy products have been found to produce bacteriocins (e.g., lactocins and enterocins) [94,95]. In the present study, none of the isolates showed antimicrobial activity against *L. innocua* (Table 3). However, the ability of lactic acid bacteria to synthesize bacteriocins is strictly strain-dependent and is affected by the expression of numerous genes [93].

## 4. Conclusions

The aim of this study was to isolate and characterize lactic acid bacteria from *viili* fermented milk samples obtained from natural starter cultures. This study aimed to assess the technological and safety traits of the isolates to identify potential candidates suitable for use as starter or adjunct cultures in dairy products.

Based on the results obtained in the present study, the isolates *L. cremoris* 1.12 and *L. lactis* 2.15 exhibited promising pro-technological traits (such as acidification performance and protease activity) for their use as starter cultures. Isolate 1.12 also showed leucine arylamidase, beta-galactosidase, and beta-glucosidase activities. Additionally, isolate 2.15 exhibited a positive reaction for acid phosphatase and naphthol-AS-BI-phosphohydrolase. Several *E. faecium* and *E. faecalis* isolates, namely 1.13, 1.14, 1.15, 2.1, and 2.2, showed significant lipase activity, a key feature for their potential use as adjunct cultures for flavor enhancement of the product. However, the presence of *Enterococcus* spp. in dairy products raises safety concerns due to virulence traits and antibiotic resistance occurring in certain strains.

Despite the observed slimy texture in the *viili* fermented milk samples studied herein, which suggested the presence of EPSs in the end product, no isolate exhibited the in vitro production of these natural biopolymers. Consequently, further investigation is needed to test the selected microbial cultures in in vivo *viili* models. Furthermore, future research should focus on investigating the presence and function of fungal populations in *viili*.

Of note, the detection of the *hdcA* gene in isolates 1.5, 2.7, 2.11, 3.13, and 4.14 highlights their unsuitability for dairy processing and sheds a first light on the potential histamine risk associated with the consumption of this fermented food.

Overall, the findings of this study have contributed to the identification and selection of lactic acid bacteria from artisanal *viili,* thereby enhancing the preservation of the quality and authenticity of this product. Moreover, several cultures of *L. cremoris* and *L. lactis* exhibited promising features for their application in dairy processing.

## Figures and Tables

**Table 1:** pH, titratable acidity, and counts of presumptive lactic acid bacteria monitored during the activation of *viili* natural starters.

	pH				Titratable Acidity ^1^				Presumptive Lactic Acid Bacteria ^2^			
	V1	V2	V3	V4	V1	V2	V3	V4	V1	V2	V3	V4
t_0_	6.49 ± 0.07 ^A^	6.35 ± 0.07 ^A^	6.48 ± 0.07 ^A^	6.55 ± 0.07 ^A^	0.14 ± 0.01 ^D^	0.16 ± 0.01 ^C^	0.12 ± 0.01 ^C^	0.14 ± 0.01 ^C^	1.85 ± 0.00 ^d,D^	2.68 ± 0.23 ^c,C^	3.95 ± 0.11 ^a,B^	3.26 ± 0.02 ^b,C^
t_1_	6.18 ± 0.01 ^a,B^	6.11 ± 0.01 ^a,B^	4.53 ± 0.01 ^c,B^	5.26 ± 0.01 ^b,B^	0.15 ± 0.01 ^c,D^	0.18 ± 0.01 ^c,B,C^	0.62 ± 0.00 ^a,B^	0.42 ± 0.01 ^b,B^	7.51 ± 0.05 ^c,C^	6.29 ± 0.06 ^d,B^	9.25 ± 0.04 ^a,A^	8.78 ± 0.07 ^b,B^
t_2_	5.81 ± 0.01 ^a,C^	5.77 ± 0.00 ^a,C^	4.30 ± 0.01 ^b,C^	4.34 ± 0.01 ^b,C^	0.25 ± 0.03 ^b,C^	0.24 ± 0.01 ^b,B^	0.74 ± 0.03 ^a,A^	0.76 ± 0.02 ^a,A^	8.99 ± 0.00 ^b,A^	9.55 ± 0.06 ^a,A^	9.58 ± 0.03 ^a,A^	9.60 ± 0.01 ^a,A^
t_3_	4.70 ± 0.01 ^a,D^	4.28 ± 0.01 ^b,D^	4.32 ± 0.00 ^b,C^	4.25 ± 0.01 ^b,C^	0.55 ± 0.02 ^b,B^	0.72 ± 0.03 ^a,A^	0.74 ± 0.02 ^a,A^	0.76 ± 0.02 ^a,A^	8.93 ± 0.01 ^b,A^	9.59 ± 0.14 ^a,A^	9.48 ± 0.01 ^a,A^	9.56 ± 0.09 ^a,A^
t_4_	4.40 ± 0.01 ^E^	4.30 ± 0.00 ^D^	4.37 ± 0.01 ^C^	4.27 ± 0.01 ^C^	0.73 ± 0.02 ^A^	0.74 ± 0.02 ^A^	0.73 ± 0.01 ^A^	0.76 ± 0.05 ^A^	8.49 ± 0.02 ^b,B^	9.43 ± 0.02 ^a,A^	9.43 ± 0.14 ^a,A^	9.53 ± 0.02 ^a,A^

t_0_: milk immediately after inoculation; t_1_: inoculated milk after incubation; t_2_, t_3_, t_4_: inoculated milk after the first, second, and third sub-culturing steps. Values are expressed as mean ± standard deviation of duplicate independent measurements. Within each row, means with different lowercase superscript letters are significantly different (*p* < 0.05). Within each column, means with different uppercase superscript letters are significantly different (*p* < 0.05). ^1^ Expressed as % of lactic acid equivalents. ^2^ Expressed as Log cfu g^−1^.

**Table 2:** Identification of lactic acid bacteria isolated from activated *viili* natural starters.

Isolation Source	Isolate Code	Closest Relative	% Identity *	Accession Number **
V1	1.1	*Enterococcus lactis*	99.25%	NR_117562
	1.2	*Enterococcus lactis*	99.30%	NR_117562
	1.3	*Enterococcus faecium*	99.33%	NR_113904
	1.4	*Enterococcus faecium*	99.37%	NR_113904
	1.5	*Enterococcus faecium*	99.61%	NR_113904
	1.6	*Enterococcus durans*	99.58%	NR_036922
	1.7	*Enterococcus faecium*	99.35%	NR_113904
	1.8	*Lactococcus cremoris*	99.71%	NR_040954
	1.9	*Lactococcus cremoris*	98.97%	NR_040954
	1.10	*Lactococcus cremoris*	99.01%	NR_040954
	1.11	*Lactococcus cremoris*	99.81%	NR_040954
	1.12	*Lactococcus cremoris*	98.99%	NR_040954
	1.13	*Enterococcus faecium*	99.25%	NR_113904
	1.14	*Enterococcus faecium*	99.79%	NR_113904
	1.15	*Enterococcus faecium*	99.48%	NR_113904
V2	2.1	*Enterococcus faecalis*	99.64%	NR_115765
	2.2	*Enterococcus faecalis*	99.53%	NR_115765
	2.3	*Enterococcus durans*	99.58%	NR_036922
	2.4	*Enterococcus faecalis*	99.22%	NR_115765
	2.5	*Lactococcus lactis*	99.16%	NR_040955
	2.6	*Lactococcus lactis*	99.16%	NR_040955
	2.7	*Lactococcus lactis*	99.66%	NR_040955
	2.8	*Lactococcus lactis*	99.87%	NR_040955
	2.9	*Lactococcus lactis*	99.57%	NR_040955
	2.10	*Lactococcus lactis*	98.85%	NR_040955
	2.11	*Lactococcus lactis*	99.68%	NR_040955
	2.12	*Lactococcus lactis*	99.48%	NR_040955
	2.13	*Lactococcus lactis*	99.36%	NR_040955
	2.14	*Enterococcus durans*	99.34%	NR_036922
	2.15	*Lactococcus lactis*	99.62%	NR_040955
V3	3.1	*Lactococcus lactis*	99.30%	NR_040955
	3.2	*Lactococcus lactis*	99.84%	NR_040955
	3.3	*Lactococcus lactis*	99.71%	NR_040955
	3.4	*Lactococcus lactis*	99.26%	NR_040955
	3.5	*Lactococcus lactis*	99.75%	NR_040955
	3.6	*Lactococcus lactis*	99.72%	NR_040955
	3.7	*Lactococcus lactis*	99.41%	NR_040955
	3.8	*Lactococcus lactis*	99.57%	NR_040955
	3.9	*Enterococcus faecalis*	99.33%	NR_115765
	3.10	*Enterococcus faecalis*	99.85%	NR_115765
	3.11	*Enterococcus faecalis*	99.12%	NR_115765
	3.12	*Lactococcus lactis*	99.71%	NR_040955
	3.13	*Lactococcus lactis*	99.87%	NR_040955
	3.14	*Lactococcus lactis*	99.61%	NR_040955
	3.15	*Lactococcus lactis*	99.49%	NR_040955
V4	4.1	*Enterococcus durans*	99.23%	NR_036922
	4.2	*Enterococcus durans*	99.29%	NR_036922
	4.3	*Enterococcus durans*	99.42%	NR_036922
	4.4	*Enterococcus durans*	99.00%	NR_036922
	4.5	*Lactococcus lactis*	99.54%	NR_040955
	4.6	*Lactococcus lactis*	99.86%	NR_040955
	4.7	*Lactococcus lactis*	99.19%	NR_040955
	4.8	*Lactococcus lactis*	99.26%	NR_040955
	4.9	*Lactococcus lactis*	99.70%	NR_040955
	4.10	*Lactococcus lactis*	100.00%	NR_040955
	4.11	*Lactococcus lactis*	99.61%	NR_040955
	4.12	*Lactococcus lactis*	99.88%	NR_040955
	4.13	*Lactococcus lactis*	99.15%	NR_040955
	4.14	*Lactococcus lactis*	99.73%	NR_040955
	4.15	*Lactococcus lactis*	99.00%	NR_040955

* Percentage of identical nucleotides in the sequence obtained from the bacterial isolates and the sequence of the closest relative found in the GenBank database. ** Accession number of the sequence of the closest relative found by BLAST search.

**Table 3:** Characterization of lactic acid bacteria isolated from *viili* samples.

Isolate Code	Closest Relative	Protease Activity *	Lipase Activity *	Esterase Activity *	EPS Production	*hdcA* Gene	Antimicrobial Activity
1.1	*Enterococcus lactis*	+	+	-	-	-	-
1.2	*Enterococcus lactis*	+	-	-	-	-	-
1.3	*Enterococcus faecium*	++	-	-	-	-	-
1.4	*Enterococcus faecium*	+	-	-	-	-	-
1.5	*Enterococcus faecium*	+	+	-	-	+	-
1.6	*Enterococcus durans*	-	-	-	-	-	-
1.7	*Enterococcus faecium*	++	-	-	-	-	-
1.8	*Lactococcus cremoris*	+	-	-	-	-	-
1.9	*Lactococcus cremoris*	+	-	-	-	-	-
1.10	*Lactococcus cremoris*	+	-	-	-	-	-
1.11	*Lactococcus cremoris*	+	-	-	-	-	-
1.12	*Lactococcus cremoris*	++	-	-	-	-	-
1.13	*Enterococcus faecium*	+	++	-	-	-	-
1.14	*Enterococcus faecium*	+	++	-	-	-	-
1.15	*Enterococcus faecium*	+	++	-	-	-	-
2.1	*Enterococcus faecalis*	+	++	-	-	-	-
2.2	*Enterococcus faecalis*	+	++	-	-	-	-
2.3	*Enterococcus durans*	+	-	-	-	-	-
2.4	*Enterococcus faecalis*	+	-	-	-	-	-
2.5	*Lactococcus lactis*	+	-	-	-	-	-
2.6	*Lactococcus lactis*	+	-	-	-	-	-
2.7	*Lactococcus lactis*	-	-	-	-	+	-
2.8	*Lactococcus lactis*	-	-	-	-	-	-
2.9	*Lactococcus lactis*	-	-	-	-	-	-
2.10	*Lactococcus lactis*	+	-	-	-	-	-
2.11	*Lactococcus lactis*	++	-	-	-	+	-
2.12	*Lactococcus lactis*	+	-	-	-	-	-
2.13	*Lactococcus lactis*	+	-	-	-	-	-
2.14	*Enterococcus durans*	++	+	-	-	-	-
2.15	*Lactococcus lactis*	++	-	-	-	-	-
3.1	*Lactococcus lactis*	+	-	-	-	-	-
3.2	*Lactococcus lactis*	+	-	-	-	-	-
3.3	*Lactococcus lactis*	+	-	-	-	-	-
3.4	*Lactococcus lactis*	-	-	-	-	-	-
3.5	*Lactococcus lactis*	-	-	-	-	-	-
3.6	*Lactococcus lactis*	-	-	-	-	-	-
3.7	*Lactococcus lactis*	-	-	-	-	-	-
3.8	*Lactococcus lactis*	-	-	-	-	-	-
3.9	*Enterococcus faecalis*	-	-	-	-	-	-
3.10	*Enterococcus faecalis*	+	-	-	-	-	-
3.11	*Enterococcus faecalis*	-	-	-	-	-	-
3.12	*Lactococcus lactis*	+	-	-	-	-	-
3.13	*Lactococcus lactis*	+	-	-	-	+	-
3.14	*Lactococcus lactis*	+	-	-	-	-	-
3.15	*Lactococcus lactis*	+	-	-	-	-	-
4.1	*Enterococcus durans*	+	+	-	-	-	-
4.2	*Enterococcus durans*	+	+	-	-	-	-
4.3	*Enterococcus durans*	+	-	-	-	-	-
4.4	*Enterococcus durans*	+	+	-	-	-	-
4.5	*Lactococcus lactis*	-	-	-	-	-	-
4.6	*Lactococcus lactis*	-	-	-	-	-	-
4.7	*Lactococcus lactis*	-	-	-	-	-	-
4.8	*Lactococcus lactis*	-	-	-	-	-	-
4.9	*Lactococcus lactis*	-	-	-	-	-	-
4.10	*Lactococcus lactis*	+	-	-	-	-	-
4.11	*Lactococcus lactis*	-	-	-	-	-	-
4.12	*Lactococcus lactis*	+	-	-	-	-	-
4.13	*Lactococcus lactis*	+	-	-	-	-	-
4.14	*Lactococcus lactis*	+	-	-	-	+	-
4.15	*Lactococcus lactis*	++	-	-	-	-	-

* -, negative; +, positive (1 mm halo); ++, positive (1–2 mm halo).

## Data Availability

The original contributions presented in the study are included in the article; further inquiries can be directed to the corresponding author.
